# Post-traumatic growth and cancer survivorship: experiences of living with treatment-related impairment

**DOI:** 10.1007/s00520-026-10341-6

**Published:** 2026-01-22

**Authors:** Rebecca Davis, Ruth Jones, Kerith Duncanson

**Affiliations:** 1 Oncology Department, Daffodil Cottage, Western NSW LHD, Bathurst, Australia; 2 Lifeline Central West, Bathurst, Australia; 3 Cancer Services and Innovation, Western NSW Local Health District, Dubbo, Australia; 4 NSW Health Education and Training Institute, St Leonards, Australia; 5 Hunter Medical Research Institute, Level 3 East, New Lambton Heights, NSW 2305 Australia

**Keywords:** Post-traumatic growth, Cancer, Impairment, Meaning making, Psycho-oncology

## Abstract

**Purpose:**

Increased distress has been associated with impairment related to cancer treatment and with post-traumatic growth (PTG), but the influence of treatment-related impairment on PTG has not been explored. This study aimed to understand the lived experience of PTG for cancer survivors living with treatment-related impairment.

**Methods:**

Hermeneutic phenomenology was used to develop a deep understanding of the lived experience of adult cancer survivors living with ongoing treatment-related impairment who had experienced self-perceived PTG following their cancer experience. Semi-structured individual interviews conducted with eight participants were transcribed, manually coded, and thematically analysed.

**Results:**

This study demonstrated that people with ongoing treatment-related impairment can experience PTG through coping with cancer. PTG was both a coping process and an outcome of coping with adversity. Participants experienced growth outcomes in the domains of new possibilities, relating to others, personal strength, and appreciation of life. Participants first experienced a state of incongruence arising from the intrusion of cancer, which challenged their existing world view and self-concept. They reported using coping strategies to manage distress, enabling productive meaning making. A notable absence of distress about impairment was attributed to participants facing this later in their cancer trajectory when meaning making was well established, and their experience of impairment more readily assimilated.

**Conclusion:**

The extent of PTG in cancer survivors may depend on the degree of incongruence they experience and their ability to accommodate these contradictions to develop new meaning. Further research is needed to understand how early or visible treatment-related impairment influences cancer survivors’ PTG.

## Introduction

Improved cancer survival rates have led to increased demand for survivorship care, with the long-term quality of life of cancer survivors becoming a focus [1, 2] and increased understanding of long-term survivorship experiences warranted [3]. Research has recently shifted away from analysing purely the negative psychosocial impacts associated with the experience of cancer, with greater recognition of and interest in the positive changes that can result from a cancer diagnosis [4], including post-traumatic growth (PTG). Tedeschi and Calhoun first coined the concept of PTG, defined as “positive psychological changes experienced as a result of the struggle with major life crises or traumatic events” [5]. These authors developed the Post-Traumatic Growth Inventory (PTGI), a standardised measure to assess PTG, which includes domains of new possibilities, personal strength, relating to others, spiritual change, and appreciation of life. PTG can be viewed as both an *outcome* of coping with traumatic events and a coping *process* [5]. PTG is portrayed as a transformative meaning making process where one integrates new understandings of self into their vision for the future; a subjective process which is dependent upon emotional, cultural, and temporal characteristics [4].

Cancer trauma has some unique characteristics, including the typically chronic, intangible, and uncertain nature of the threat, and that threat being internal, or embodied [2, 6]. These characteristics are thought to influence the process of PTG and how it presents. Psychological growth is thought to emerge from the cognitive and behavioural adaptations that occur as an individual copes with their cancer experience, with the initial threat, distress, and adversity fundamental to the promotion of PTG [7]. A positive relationship between growth and stress has been demonstrated in cancer populations [2].

Increased cancer survival rates have resulted in more people living with treatment-related impairment [3, 8], which may persist or worsen over time [9]. The World Health Organization (2001) International Classification of Functioning, Disability and Health (ICF) defines impairment as “problems in body function or structure such as a significant deviation or loss” [10]. Cancer survivors experience a range of impairments that may affect any organ system [11]. The identification and management of late and long-term treatment-related effects is a fundamental yet often neglected aspect of survivorship care [9]. This issue is complicated by survivors’ tendency towards reluctance in discussing side effects and impairment, even those who have endured similar experiences [3]. The majority of survivors have significant impairment following treatment for cancer, which often goes undetected or untreated, and may result in disability [11]. It is therefore pertinent to understand the experiences of people who bear permanent or long-term treatment-related impairment.

There is emerging awareness about the presence of growth in survivors with ongoing treatment-related impairment. Personal growth, enhanced appreciation for life, and refocused priorities have been reported [12], as have the development of post-cancer identities featuring a renewed role in and commitment to personal relationships [3]. Newfound empathy for others with cancer and a new sense of identity have been described [13]. To date, this research has largely been conducted with breast cancer survivors, with a relative gap in knowledge in relation to survivors from other tumour streams [14]. Breast cancer survivors also comprise the majority of participants in PTG studies that are not confined to a specific cancer type [4, 15]. The impact of cancer type on post-diagnosis adjustment, specifically PTG, has not been comprehensively examined [16]. Although research about PTG in rural cancer populations is sparse, several US studies suggest rurality may be influential in the PTG experience [7, 17].

Despite the broadening of survivorship research to encompass positive psychosocial developments arising from the cancer experience, studies relating to treatment-related impairment are largely focused on negative consequences of treatment [8, 9], with an overarching emphasis on reduced quality of life [18, 19]. The aim of this study was to understand the lived experience of PTG for rural cancer survivors living with treatment-related impairment.

## Methods

This qualitative study adopted hermeneutic phenomenology methodology, using Van Manen’s approach which melds together processes of description and interpretation to understand the meaning of lived experience [20]. We used hermeneutic processes to make explicit what is generally implicit in human experiences and interactions to reveal embedded meaning [21], which may not have been obvious to participants yet are derived from their narrative.

### Participant sampling and recruitment

Study participants were adult cancer survivors living with treatment-related impairment living in Western NSW, Australia. Targeted recruitment involved identification of patients who met medical inclusion criteria (Table 1) by medical and allied health MDT staff. Maximum variation sampling involved choosing invitees from a range of tumour streams and impairment types. The only medical information shared with the research team was details relating to study criteria.

**Table 1:** Inclusion and exclusion criteria for study

Inclusion criteria
• Diagnosed with cancer within the previous 5 years
• Completed their primary cancer treatment (e.g. surgery or initial course of chemotherapy or radiation treatment; may or may not be currently undergoing cancer treatment)
• Currently or previously utilised oncology services through Central West Cancer Care Centre
• Experiencing treatment-related impairment that is considered permanent, highly likely to be permanent or persist for 12 months or more, according to current medical evidence
• Experienced self-perceived post-traumatic growth (as described in screening process and participant information sheet) as a result of their cancer experience
• Ability to speak and understand English to the extent that enables informed and purposeful participation
• Capacity to make their own informed decisions about the healthcare and medical treatment they receive (i.e. capacity will not be assessed, but implied if the person routinely makes their own medical and healthcare decisions and there is no legal guardian currently appointed for this purpose)
Exclusion criteria:
• Diagnosed with breast cancer as primary cancer

Invitees were mailed a recruitment letter, participant information sheet (PIS), and consent form. Invitees who wished to participate contacted the research team via phone or email, with eligibility then confirmed and an interview scheduled. Invitees who did not initiate contact were phoned after 2 weeks to address questions or decline participation. As some cancer survivors may not disclose treatment-related impairment to their treating team, recruitment flyers were also displayed in cancer centre waiting areas, with interested parties invited to contact the research team about eligibility. The same recruitment processes as for invitees were then employed. All participants provided written consent to participate.

### Data collection

Participants completed semi-structured individual interviews with the PI. Seven interviews were conducted in person and one via videoconference. Interviews were conducted in a private room in a health service facility or a mutually agreed alternate location in the community. Interviews were digitally recorded. Field notes were journalled at the time of the interview to add to the data available for analysis and for interpretive rigor. Semi-structured interviews were informed by a flexible interview guide (Table 2) of open-ended questions, complemented by follow-up questions and probes in relation to participant responses. Available literature and the chosen methodology were used to inform question development and design of the semi-structured interviews, with questions focused on examining the phenomenon as it was experienced rather than how it was conceptualised. Interview questions were piloted with a cancer survivor to ensure sensitive, survivor-centred data collection. No participants chose to review transcripts.

**Table 2:** Interview guide for study on post-traumatic growth and experiences of living with cancer treatment-related impairment

Usual sequence		Prompts and probes
1	Introduction to research and interviewer	
2	Please tell me about yourself so I can get to know you a little	
3	Where are you at in terms of your cancer treatment/care at the moment?	Cover how long since treatment, duration of treatment, type of treatment (may come up in Q2)
4	Would you please describe your experience of being diagnosed with cancer to me?	As much detail as you wish at this time. Acknowledge difficulty
5	What was the experience of having treatment for cancer like for you?	Probe on how they felt about it if response focused on what happened
6	Could you now tell me about the ongoing effects or problems you experience as a result of your cancer treatment?	
7	Can you now reflect back and then describe to me any important or defining moments that you recall from your cancer experience?	
8	Provide description of post-traumatic growth. Can you tell me about your own experience of PTG?	What do you think has helped you to experience this growth?
6	How has your (impairment- as described by participant) impacted/affected your experience of PTG?	Impairment described in Q6
7	If you think about yourself before you were diagnosed with cancer, and compare it to the person sitting here today, what changes do you see?	

General probes and prompts used to assist the participant to explore the topic/situation in more depth

• Please tell me more about what that was/felt like for you

• Can you give me an example?

• What was that experience like for you?

• How did you feel about…?

• What happened then?

• When did this happen?

• • Why do you think that is?

### Data analysis

Van Manen’s holistic approach to thematic analysis was used, with the detailed and selective techniques providing a framework for analysing the deeper meaning of the experience. The process involved initial reading of the transcripts, then line-by-line reading for familiarisation and engagement with the data. Common words and ideas were identified and manually coded, with statements of significance also identified. Through observing and interpreting the context and meaning of participant narratives, themes were identified, then refined through writing and re-writing. The PI returned to the transcripts repeatedly and had regular co-analysis discussions with co-researchers to locate, develop, and validate the final themes. Reflecting on themes maintained a strong orientation to the phenomenon of interest [20, 22].

### Reflexivity

Consistent with hermeneutic phenomenology, researchers’ biases and assumptions were considered essential to the analysis process through thoughtful consideration of our own experience in relation to the research topic [21]. Prior knowledge of the research topic is considered a pre-requisite for conducting hermeneutic phenomenology [23]. The researchers interpreted the lived experiences of cancer survivors, so their approach to practice, experience, and interest in the topic are described. The PI’s social work lens motivated the qualitative, phenomenological approach of this study, which promotes the valuing of lived experience and a belief in human potential. The research focus and methodology are grounded in a strength-based and person-centred perspective, which promotes a holistic, biopsychosocial approach to practice and a focus on the subjective elements of human experience. Throughout the process of analysis, the PI kept a reflective journal, detailing interpretations and reflections, and regularly reviewed data and analyses with other members of the research team to ensure reflexivity. The co-researchers included an experienced clinician researcher who is the director of a cancer service and a qualitative researcher with lived experience of cancer survivorship.

### Ethical considerations

This study was conducted in accordance with the Declaration of Helsinki and received ethics and governance approvals from the Greater Western Human Research Ethics Committee.

## Findings

**Fig. 1:**
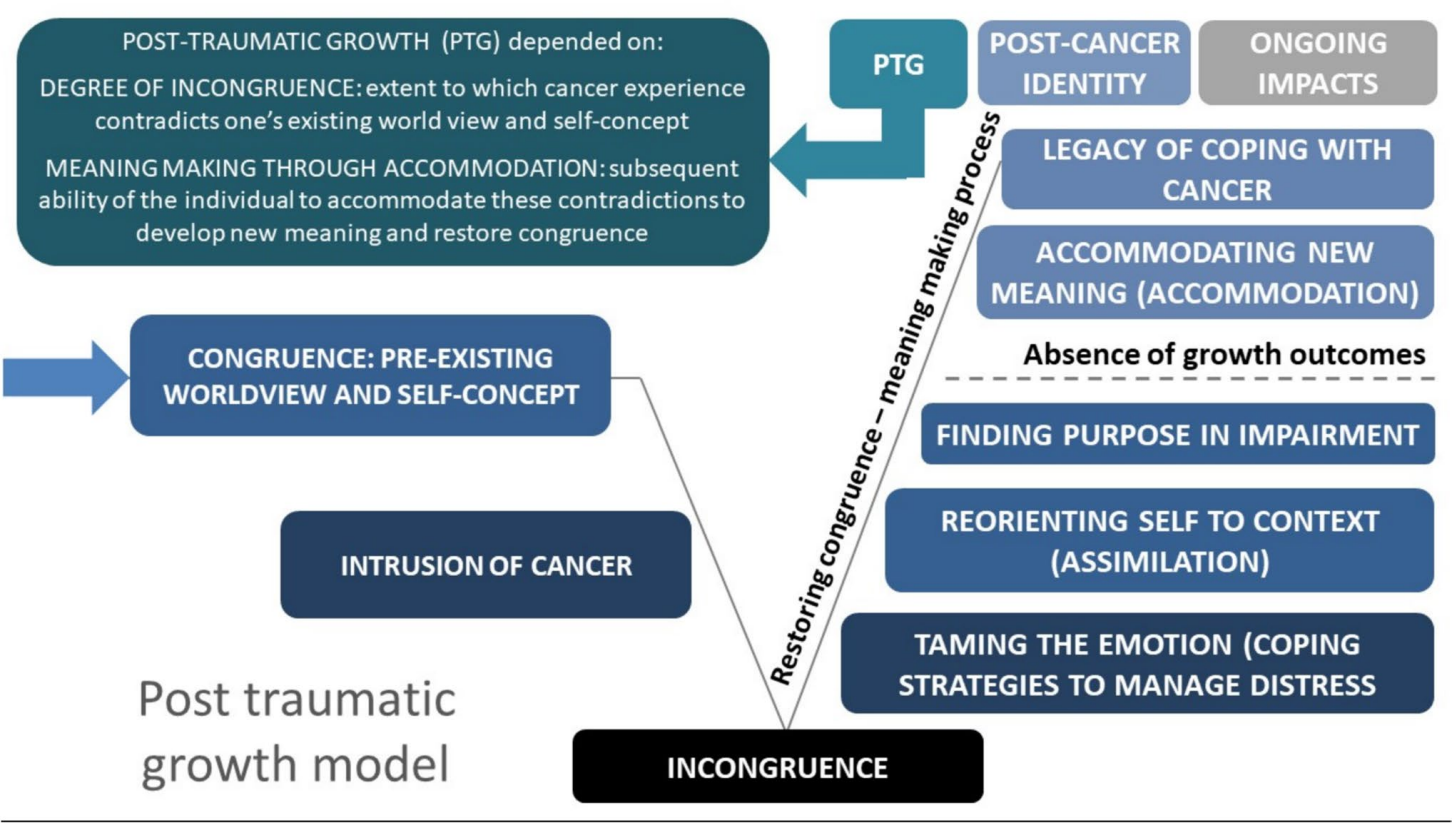
Model showing the relationship between themes and subthemes associated with post-traumatic growth in cancer survivors. Explanation of model: Cancer is a disruptive and threatening *intrusion* that causes a state of *incongruence*, where one’s existing world view and self-concept are contradicted. This state of incongruence happens to varying degrees, from feeling unsettled to a state of discombobulation or disorientation and is a precursor for experiencing post-traumatic growth (PTG). Incongruence is generally but not always accompanied by varying degrees of *distress,* which predominantly stems from feelings of powerlessness and uncertainty. Managing distress and overcoming incongruence require adaptation and orientation, which involve the *assimilation* of coping strategies that stimulate a process of *meaning making* to restore congruence. PTG emerges through this meaning making process when a relative state of congruence is restored, and new meanings are developed. The extent of incongruence and meaning making determines PTG, with distress not being a necessary precursor. Growth is most evident, tangible, and multi-faceted for people whose worldview and self-concept are contradicted in substantial and fundamental ways and who are subsequently able to resolve this incongruence through the meaning making process to develop new understandings of themselves, others, and the world around them. This is experienced as a transformative process, culminating in a distinct post-cancer identity. Whilst assimilation is a vital aspect of coping and restoring congruence, the most influential factor in determining PTG is the ability to utilise *accommodation* to achieve congruence by altering pre-existing beliefs and assumptions to match new experiences. It is possible to integrate impairment into *post-cancer identity* and achieve a sense of resolution and congruence associated with their *ongoing impairment.* When realisation that impairment is likely to be ongoing emerges in the latter stages of their cancer trajectory, the process of meaning making is well established, allowing the *legacy of coping* with cancer to be more readily assimilated

Thirty-six patients were nominated by the MDT through the targeted recruitment strategy. No participants were identified through the self-nomination strategy. Of the 36 patients nominated, 13 were not interested in participation, six were not able to be contacted or their decision confirmed, eight were not eligible (five were beyond 5 years post-diagnosis, one was not experiencing impairment, and one did not experience self-perceived PTG), and one had died between being nominated and being contacted in relation to the study. Eight eligible participants aged between 43 and 81 years completed interviews, of whom four were male and four were female. Interviews were an average of 1 h in duration, and one participant chose to have a family member present. Tumour types included head and neck (*n* = 3), colorectal (*n* = 2), gynaecological (*n* = 2), and multiple myeloma (*n* = 1). Time since diagnosis ranged from less than 1 year up to 5 years. Two participants had incurable disease and all had experienced multimodal therapy. Impairments included peripheral neuropathy, infertility, osteonecrosis of the jaw, vision loss, dysphagia, trismus, xerostomia, and speech changes. Participants’ experiences of growth are depicted in a model comprising sequential themes (Fig. 1). No completely new codes were identified by the time of analysis of the final interview.

### Theme 1: Incongruence: the intrusion of cancer

The intrusion of cancer caused participants to experience a state of incongruence, where their existing world view and self-concept were disrupted. This incongruence necessitated practical and psychological reconstruction or re-evaluation of participants’ reality, including their sense of identity, routines, goals, and priorities. It was experienced by all participants to varying degrees yet was not always proportionate to the level of intrusion reported.

For some participants, recognition of vulnerability highlighted incongruence between pre-cancer and current self, and acknowledging this prompted a new search for meaning and identity (Participant 4, Table 3). Although incongruence permeated all cancer stages, it was particularly evident in the diagnosis stage. At this early stage, it was characterised by feelings of uncertainty, disbelief, fear, and distress. For one participant, the discombobulation they felt evidenced as a dissociative experience (Participant 2, Table 3). The sudden impact of impairment was associated with additional feelings of powerlessness, especially if treatment resulted in immediate functional limitations or conceptions of self that were deeply incongruous with pre-cancer identity (Participants 2 and 4, Table 3).

**Table 3:** Substantiating quotes relating to incongruence: the intrusion of cancer

Participant	Aspect of incongruence/subtheme	Substantiating quote
4	Recognition of vulnerability highlighted incongruence	*That was probably the turning point for me…I was really struggling to get the pace and walk up hills. I suddenly realised that I’m not the person I used to be. That was a good thing….I started to think, “well, this is the new me”*
6	Incongruence was particularly prominent in diagnosis stage	*I was a mess at the beginning when I got the diagnosis and it was terrifying, …. I couldn’t even rationalise it in my head. Like it was like “is this happening to me or is it happening to someone else?..….., they must’ve made a mistake, this can’t be happening. It was so surreal, it was like I was looking at myself outside of myself kind of thing, and I couldn’t speak to anyone about it because I was just far too emotional*
4	The sudden intrusion of cancer was associated with a sense of powerlessness, especially if cancer was deeply incongruous with self-identity	*…72 years old. I’ve been really fit all my life, extremely fit, …. I thought my life was pretty well mapped out. I didn’t expect to get this. It certainly came out of the blue. Everything I’ve ever done, I’ve been lucky I’ve been able to do well. Then suddenly I can’t*
2	Uncertainty about the future endured from diagnosis to surveillance stages	Well I feel like I was probably a bit sheltered prior…..thinking it so far removed and then it happens and that smacks you in the face and it’s like – excuse my language – it’s like f**k, that’s me…..I didn’t want anyone to know about it. I was embarrassed and I didn’t want to be that person were people look at you and go you’ve got cancer or thinking they knew your story and they didn’t and assuming things. Because I’m doing enough assuming myself
1	Incongruence was sometimes mitigated by life perspective	*I always knew something was going to bite me in the butt one day as I got older, and this is it. Just roll with the punches, so to speak. Not worry about it or dwell on it or any of that sort of thing*
5	Cancer less incongruous in light of previous cancer diagnoses or life experiences that involved risk-taking behaviours	*Yeah, a little bit scary because when I first got diagnosed with lung cancer, I said “me number’s up, so I’m going to have a wake before I go”….then after the surgery the cancer was gone, I said “Sorry boys, there’s no wake”. Later when the tongue (cancer) come along, even me mates said “we didn’t think you were going to pull through” because I was such a mess. I have been close to death a few times, closer than this. That was through stupidity*
7	Fear associated with incongruence can be mitigated by cancer survivorship	*I think it’s the fear that cancer puts on people to a certain extent…I’m not as fearful now of the situation, the word, as I would have been before. I think there’s something there because (my husband) also died of cancer*
3	Pragmatism clearly distinguished incongruence from distress	*Well, everything’s changed, because I was a full on person doing everything, going to a person that had to be grounded when I had the operations, and then I coped really well with the first lot of chemo, and then it wasn’t till the middle of that first lot that I started to get neuropathy in my hands and my feet, and then I found that really hard, not stressful, or I wasn’t anxious or anything like that. I just learned to cope with what my boundaries were…*
8	Distress was heightened if accompanied by a sense of injustice and loss, especially with treatment-related impairment	*But it was sort of a period in my life that sort of missed, missed a lot of stuff………I just broke down and cried, you know. I said to her that this shouldn’t happen to me,…. it takes your whole living away straight away, bang…… I think the crying helped me a little bit. I’ve never cried in my life for years*
7	Distress due to treatment-related preventative measures resulted in coping mechanisms such as social withdrawal to manage distress	*I had 10 good teeth taken out, which I found more distressing than anything, having kept them for 80 years, looked after them and then they’re ripped out…I couldn’t speak, I couldn’t swallow, I couldn’t eat. It all became very difficult and very stressful at that stage. But not being able to communicate, I found that one of the hardest things I’ve ever had to deal with in my life*

Incongruence was sometimes mitigated by life perspective. One participant viewed health challenges as synonymous with ageing and life events to be relatively pre-determined and uncontrollable (Participant 1, Table 3). Similarly, a potentially life-limiting illness was not considered vastly incongruous by a participant who had a previous cancer diagnosis and believed their previous risk-taking behaviours and lifestyle choices were not synonymous with longevity (Participant 5, Table 3).

Together, these findings support the proposition of Morris and Shakespeare-Finch that subjective appraisal of cancer severity rather than disease stage or objective determination of the threat may predict PTG [16]. The experiences reflect the research of Picione et al. (2017) who suggest that cancer disrupts one’s relationship with time and their sense of continuity in life. This can create feelings of uncertainty and disorientation, interrupting one’s ability to connect with past experiences.

A pragmatic approach to managing limitations and the need for adaptation clearly distinguished incongruence from distress (Participant 3, Table 3). However, distress was more prominent for some participants, mainly stemming from feelings of powerlessness and uncertainty. Most participants’ cancer diagnosis evoked a fear or expectation of death (Participant 2, Table 3). The connection between cancer and death was reinforced for some by a previous diagnosis or having a close family member experience cancer, but had eased with their own cancer survival. The distress of diagnosis was heightened if accompanied by a sense of injustice and loss, especially if it resulted in treatment-related impairment (Participant 8, Table 3). Distress resulting from treatment effects sometimes resulted in coping mechanisms such as social withdrawal, and was heightened when the treatment impacted perception of self (Participant 7, Table 3).

Based on the experiences of participants in this study, we propose that distress and growth co-occur and overlap rather than existing exclusively or in a causal relationship. Previous studies involving cancer survivors have described a range of associations between distress and PTG, some demonstrating no relationship and others demonstrating a strong positive correlation [16]. However, there is consensus that for PTG to occur, the event needs to be significant enough to shift fundamental assumptions [16], a consensus supported by this study’s findings about incongruence. Our study also suggested that distress accompanying or preceding PTG was related to specific aspects of the cancer journey that participants could define, address, or reconcile. Overcoming distress was part of their PTG journey.

The concept of incongruence is embraced within some established theories of PTG and meaning making. For example, meaning theory suggests that people hold a “global meaning” which is their own personal life framework grounded in established beliefs. This global meaning provides a sense of stability, structure, and purpose. Cancer diagnosis dismantles this framework, bringing uncertainty and instability. Future plans and goals may no longer seem attainable. The subsequent search for new meaning is a necessary yet often distressing process where the individual attempts to understand their experience and what it means for them [24]. The term “existential plight” originally posited by Weisman and Worden is pertinent to the field of oncology [24]. It describes the increased rumination about one’s existence and the possibility of nonexistence, common following a cancer diagnosis. Lee suggests that distress is relative to subjective appraisal of the threat that cancer imposes rather than the objective prognosis [24]. Tedeschi and Calhoun suggest a positive association between stress and growth, with heightened stress considered necessary to initiate a process of meaning making [5].

In summary, theme 1 frames cancer as an *intrusion* which is both disruptive and threatening. The intrusion of cancer causes a state of *incongruence*, where one’s existing world view and self-concept are disrupted and contradicted. This state of incongruence was experienced by all participants in varying degrees, from feeling unsettled to a state of discombobulation or disorientation and was identified as a precursor for experiencing post-traumatic growth (PTG). Incongruence was generally but not always accompanied by varying degrees of distress. When distress was experienced, it predominantly stemmed from feelings of powerlessness and uncertainty. Managing incongruence and distress necessitated adaptation and orientation.

### Theme 2: Restoring congruence: the meaning making process

Experiencing incongruence stimulated the meaning making process which helped restore the state of congruence. Meaning making incorporates multiple processes to manage distress arising from incongruence and understand one’s experience and what it means for them. This ruminative process is prompted by conflict between one’s perceptions about the stressful experience and their existing beliefs and perspectives. Meaning making can resolve this conflict and involves changing cognitions through processes of assimilation and accommodation [25]. Restoring congruence comprised three subthemes: “Taming the emotion”, “Reorienting self to context”, and “Finding purpose in impairment”.

#### Subtheme 2a: Taming the emotion

Participants used multiple coping strategies to manage distress and incongruence throughout their cancer experience. Emotion-focused coping strategies were commonly utilised by participants in this study to reduce, eliminate, or simply tolerate one’s emotional response to a stressor [26]. These strategies included accepting the uncontrollable, experiential avoidance, social support, keeping occupied, and positive reframing. Self-awareness of personality traits, strengths, and needs influenced participants’ ability to employ coping strategies they needed at any particular time.

An important factor in emotion taming was accepting the uncontrollable, which involved a pragmatic acceptance of the stressor being outside of one’s control. Participants emphasised a commitment to coping, a necessary step in working towards a processed acceptance that comes through a reflective meaning making process. This notion is consistent with the work of Shakespeare-Finch and Copping who emphasise acceptance of the stressful experience or trauma as fundamental to facilitating PTG [27]. For some participants, this commitment to acceptance appeared to be an initial step in the meaning making process that enabled them to access other coping strategies (Table 4). For others, accepting the uncontrollable was associated with experiential avoidance, as they reasoned that rumination was futile and impractical. This way of coping appeared to be a long-established, or default, response for these participants when faced with adversity (Table 4). This coping strategy is similar to stoicism, used by prostate cancer survivors post-diagnosis in a qualitative study by Mazariego et al. [3]. Stoicism involved disengaging from cancer-related issues to achieve a sense of control and acceptance. Accepting the uncontrollable allowed for focus on controllable aspects of one’s situation (Participant 3, Table 4).

**Table 4:** Substantiating quotes relating to taming the emotion

Participant	Aspect of taming the emotion	Substantiating quote
2	Commitment to acceptance was an initial step in meaning making process	*I was like “I don’t think I can do this. How am I going to get through this?”…and then I was like, “well this is just a case of what is going to happen I guess” and it was just trying to change my mindset*
1	Accepting the uncontrollable instead of ruminating	*As for coping, I’ve had no choice really. If I wanted to get better, I had to deal with the treatment and go with it….no good worrying about it*
3	Acceptance allowed for focus on controllable aspects of one’s situation	*The second time (recurrence), I just knew that, in my heart, that there was something that wasn’t right, but then I just took it as it came. Nothing can change what’s happened, you just do the drugs the best that you can*
6	Avoidant coping strategies did not prevent or limit growth when used selectively and in combination with active coping strategies	*But once the treatment started I just was so focused on getting through the day to day, I didn’t have the energy for any of that extra anxiety stuff… I thought it was like being in the army, it was like being?*
4	Experiential avoidance strategies used throughout life transferred to coping with cancer	*There’s always difficult jobs to do and they say “He doesn’t have a heart”. I did but I just removed myself. I think because of that experience at work …. I can remove myself and compartmentalise the cancer. Whether that’s a good thing or not I don’t know but it works for me*
8	Experiential avoidance used to maintain an optimistic outlook following cancer diagnosis	*I knew nothing, and I googled nothing about cancer in my neck and I googled nothing about radiation ever since I’ve had the problem. I just didn’t want to find…… I prefer to go and look at how many people live in Peru or somewhere, you know, rather than look at my problems*
3	Social support beneficial in coping and growth	*I’m blessed to have such a loving, caring family. Nobody would know that there was anything wrong with me unless I say…because people treat me in a normal way. They all bring comfort to me in just little ways, which is good*
1	Emotional and practical benefits from social support	*I’ve got a good boss who’s been very supportive while I was having the treatment…He kept dragging me back to work, even though I wasn’t doing a great deal. Most of the time I would just be standing around. It’s been good*
6	Support from friend diagnosed with cancer at a similar time	*Interestingly, I had a friend who was diagnosed with breast cancer around the same time and so we spoke to each other quite a lot…[we were] each other’s kind of cheer squad…..within ourselves had a kind of positive outlook perhaps and then we could also reflect that to each other as well*
3	Conscious engagement in distracting activities to cope with treatment-related pain and limitations	*“takes my thoughts away from what’s going in my body”*
6	Distraction encompassed mindfulness and relaxation	*…a lot of other times it was just too confronting and so I’d have to do that mindfulness stuff to try and disappear…*
1	Working provided a sense of purpose and relief from distress	*Just go to work. It’s (peripheral neuropathy) with me 24/7…takes my mind off it a lot. Even though you’re feeling it, you’re not thinking about it*
4	Meaningful activities used in response to the limitations of treatment-related impairment and disease progression	*I had to have something that kept me occupied….. because I couldn’t do much in the way of physical work. But luckily my other hobby is film photography, but you could have something to interest you*
2	Positive reframing was an effective means of coping throughout cancer experience, including to manage fear of recurrence	*But the doctor has said to me “I expect you to live a long and healthy life”. Not everyone gets that conversation I imagine. I’ve had the help and I’ve got people around me that love me. If you didn’t have that support and you didn’t have people that love you then how do you get through it? That’s scary*
4	Positive reframing was an effective means of reframing to uphold self-worth	*Sometimes I feel that I’m not as worthy as I used to be but I try and dismiss those thoughts because as my GP said, a lot of people, 72, aren’t doing what you’re doing anyway*
5	Positive reframing to cope with the ongoing impairment associated with treatment	*With cancer there’s no age group…You can die of cancer at 1 year old or 100-year-old…… I’m just happy to be still around anyway…gives an incentive to just keep going and knowing there’s people worse off than what I am*
6	Positive reframing created a sense of predictability and self-belief in capacity to cope with current and future challenges	*With each round of chemo, on one level it was harder to keep going back knowing how sick you were going to get, you had to be really brave and tough…..and there was that sense of, oh, I got to knock on the door to go into this place I don’t want to go. But on the other hand, it’s like you’ve done it once, or you’ve done it twice, or you’ve done it three times, you’ve only got two more to go, come on you can do it. So that constant kind of talking…*

Experiential avoidance was used by participants purposefully, in conjunction with active coping strategies. Although considered useful in alleviating distress in the short term, avoidant coping has been linked to higher distress and poor adaptation to cancer [15] and has shown a negative relationship with PTG [16]. Our study challenges these findings, as participant’s use of avoidant coping strategies did not appear to prevent or limit growth when used selectively and in combination with active coping strategies. Experiential avoidance was likened to “*going into battle*” by participant 6, with avoidance serving as a means of rationing emotional resources and maintaining regimented routines (Participant 6, Table 4). Participants also recognised that experiential avoidance strategies of dissociation that had been used to cope with childhood trauma or compartmentalising emotions in challenging work roles were transferred to coping with cancer (Participant 4, Table 4). Another way that experiential avoidance was employed was to maintain an optimistic outlook following cancer diagnosis. One participant consciously limited his access to information about his diagnosis and treatment to manage the high distress, an approach that contradicted his natural inclination to research matters that were unfamiliar to him. He acknowledged that the unprecedented circumstances necessitated a change in response (Participant 8, Table 4).

Social support was identified by all participants as beneficial in their coping and growth, and was provided by family, friends, work colleagues, and fellow cancer survivors. Social support from family and colleagues was considered to promote dignity of risk and independence, maintain normality, provide empathy, and shared understandings of cancer. Family was viewed as a motivation, providing a sense of purpose and gratitude (Table 4). Participants reflected that they had experienced emotional and practical benefits from social support, which ranged from ongoing employment to peer support from fellow cancer patients. The association between social support and PTG evident in this study is consistent with the well-established role of social support as a determinant of PTG [6, 28], particularly for the PTG domains of “new possibilities” and “relating to others” [14]. Social support is a valuable source of meaning and connection [29] and is thought to promote emotional expression and cognitive processing of the event, which enables growth [16].

Participants reported that keeping occupied provided valuable distraction, normality, and fulfilment. It conferred a sense of relief and purpose to help combat the incongruence they experienced. Engaging in activities for distraction is an ongoing strategy to cope with pain and limitations associated with treatment-related impairment (Participant 3, Table 4). For some participants, distraction encompassed mindfulness and relaxation techniques to cope with treatment-related distress, while for others, working provided relief from distress while maintaining a sense of purpose (Table 4). Meaningful activities were also intentionally used in response to the limitations of treatment-related impairment and disease progression. Distraction has been similarly highlighted as a productive coping strategy post-diagnosis for men with prostate cancer in Mazariego et al.’s qualitative study as distracting activities indicated a return to normalcy and that they were progressing forward with life [3].

Positive reframing was a highly utilised coping strategy and fundamental to participants’ meaning making process. Positive reframing involved participants modifying their perceptions of their experience to manage incongruence and distress. Participants who utilised positive reframing tended to draw upon it repeatedly throughout their cancer experience. Participant 2 framed this process as trying to “*keep my chimney open*”, describing her endeavour to maintain logical thoughts amid high emotion. Participants utilised positive reframing to encourage themselves to persist through challenging treatment experiences, to find gratitude in aspects of their medical and personal circumstances, to uphold their existing self-worth and self-identity, to reaffirm belief in their coping capacities, and to create a sense of predictability and hope.

Positive reframing has consistently been associated with cancer populations experiencing PTG [3, 6, 30], enabling people to attribute new meanings to adverse events, supporting adaptation and development of personal strength [1]. In our study, positive reframing was considered an effective means of coping throughout the whole cancer experience, including to manage fear of recurrence (Participant 2, Table 4). Positive reframing was also highlighted as an effective means of re-interpreting the situation and upholding a sense of self-worth in the difficult transition from pre-cancer to current self (Participant 4, Table 4). Use of positive reframing to cope with the ongoing impairment associated with treatment grounded participants with gratitude and motivation, which was crucial to experiencing PTG and consequent appreciation for life (Table 4). Participant 6 drew on positive reframing to create a sense of predictability and self-belief in her capacity to cope with current and future challenges. This was described as a constant process of reappraisal, involving self-talk, self-validation, and purposeful rumination about her circumstances as well as reflection on her coping (Table 4).

Emotion-focused coping strategies are known to be helpful when circumstances are outside of one’s direct control [14], so it is comprehensible that this style of coping would feature heavily in discussion about experiences of coping with cancer in our study. Ahadi et al. (2013) compared the coping styles of cancer patients to the general population and found the two groups demonstrated distinct coping styles [26]. They found that cancer patients relied predominantly on emotion-focused coping strategies and utilised problem-focused coping to a lesser extent compared to healthy people. Ahadi et al. contextualise their findings using Lazarus-Folkman’s behaviour model, which views coping as dynamic and context dependent, and in instances where stress is viewed as uncontrollable, emotion-focused coping is used in preference of problem-focused coping.

Problem-focused coping, which relates to active efforts to modify or eliminate the source of stress directly and is used primarily when a person considers a stressor within their capacity to change [26], was used only minimally by participants. Participants’ use of problem-focused coping was limited to seeking advice, information, and professional input in relation to their cancer. The limited use of problem-focused coping is consistent with other research which indicates problem-focused coping strategies are unproductive in managing emotions in situations that are uncontrollable [15, 26]. The current study supports the findings of Morris et al., in that drawing on a combination of problem-focused and emotion-focused coping strategies is associated with the perception of positive life changes [14]. The present study also supports existing research which suggests that drawing upon multiple strategies for coping is more helpful than relying on a single coping strategy [14, 26]. The role of both active and avoidant coping in the meaning making process is supported by Joseph and Linley, who acknowledge the processing of trauma-related information is characterised by fluctuating between states of avoidance and confrontation [31].

This subtheme advances the notion that management of psychological distress is crucial in supporting one’s capacity to cognitively process the experience to enable growth [28]. This notion is also central to the work of Tedeschi and Calhoun, who propose that if stress is not effectively managed and becomes overwhelming, the opportunity for growth is restricted as the person is not able to effectively process their experience [5]. They posit that it is essential to manage emotional distress in the early stages post-trauma, a notion that is overwhelmingly reinforced in the experiences of study participants.

#### Subtheme 2b: Reorienting self to context: assimilation provides a vision for coping

Participants engaged in a process of meaning making which involved reorienting themselves to their context in response to the incongruence created by their cancer experience. Participants assimilated long-established, stable aspects of self, including their worldview, values, and life experiences, to contextualise their cancer experience. Their new experiences of coping with cancer were incorporated into the pre-existing perspectives and experiences, as participants sought to restore congruence. Pre-existing worldviews acted like a compass, orienting them towards the best way to respond and providing them with a vision for coping. Participants demonstrated a tendency to seek out what was already known and familiar to them and apply their past experiences of coping with adversity to their coping with cancer.

Previous experiences of adversity were influential in participants’ experience of PTG in this study. One participant reflected that her tough upbringing bred resilience and had led her to view life as inherently challenging. She prided herself on being resilient and positive in the face of adversity, and expressed a strong intention to remain consistent with this sense of self through future challenges, including her incurable diagnosis. This process of meaning making appeared to both facilitate acceptance of her prognosis and provide a sense of comfort and stability. An alignment to her identity, values, and worldview provided a buffer against incongruence and distress associated with her cancer experience (Participant 3, Table 5):

**Table 5:** Substantiating quotes relating to reorienting self to context

Participant	Aspect of incongruence/subtheme	Substantiating quotes
3	Upbringing bred resilience, which supported positivity in the face of adversity	*I think that my upbringing when I was young, because it was really hard, was a lot to do with coping with things that are hard………..Well, I look at life as a challenge and a journey, so then I just take it all, as I said, as it all comes, and I can’t explain it in any other way* *Well, right from the start, I had no negativity, and I don’t have any of that now. I’ve kept positive all the way along, and that’s been beneficial for me, and that’s how that I’ll keep going right till the end sort of thing*
7	Strong self- and collective-identity from kinship ties	*I think as a family, my parents went through the war. They were teenagers in the depression and those things still carry on…..We’re a family of doers and get on and deal with it and we’ve all been a bit like that (disabled grandchild’s) birth was another thing … it shocked us and rocked us and we had to deal with that. Then, I’ve got arthritis quite badly…..and I think, all those things add to make life what it is* *I’m not afraid of challenges and I think that’s something and I think with the cancer that was a help*
4	Life stage helped assimilation	*If I was 50 it would be very, very different…I would have expected another 20 years where I could do everything myself. But you’ve got to accept that as you get older there’s certain things you can’t do*
1	Pragmatic approach to life applied to cancer experience, facilitating assimilation	*No big changes. It is what it is. Just get on and deal with it, handle it sort of thing……Keep going as I am now. Keep working until I’m about 70 or so or 80 or whatever*

The notion of identity at both the individual and collective levels was also evident from interviews. Participant 7 viewed herself, her family, and her generation as proactive in confronting adversity and addressing challenges through practical problem solving and adaptation. She expressed a sense of shared identity and belonging in these descriptions and referred to intergenerational factors which have impacted her worldview and response to adversity. Participant 4 similarly reflected on how his age and life stage helped to assimilate his experience and restore congruence (Table 5).

Participant 1’s stoic and practical approach appeared consistent with his self-concept and worldview. He utilised familiar ways of coping and implied that he was determined to remain unchanged from his cancer experience. This approach favoured the use of assimilation over accommodation (Participant 1, Table 5). This sense of subjective wellbeing through overcoming cancer while using assimilation to maintain alignment with worldview and self-concept is consistent with the previously reported association between reinforcement of pre-existing beliefs through assimilation and feelings of improved wellbeing, although lack of resolution often remains [32].

Older age was consistently identified as an enabling factor in coping, serving to reduce incongruence and distress, aid acceptance of their new reality, and support assimilation of their cancer experience. Additionally, younger age was emphasised as a factor which led to increased incongruence and distress, previously viewing cancer as a disease impacting older people. Moye et al. also reported that older cancer survivors drew on previous experiences of coping with life adversity, with increased age associated with resilience and reduced impact of events [25].

Participants’ utilisation of assimilation, including gravitating towards established aspects of self and environment, reflects a common experience in times of trauma or change. Cancer poses a threat to one’s physical and psychological wellbeing and influences the way in which one perceives themselves, their environment, and their interactions within it. This threat is the common basis upon which stress and growth are experienced in cancer populations. The cognitive process of assimilation, along with accommodation, is the means through which adjustment to a threatening experience occurs. Assimilation manages the stressful event by incorporating it into one’s pre-existing beliefs to prevent them from changing. Assimilation typically appears in the aftermath of the trauma or stress and can be helpful to reduce stress during periods where the threat is still present [33], which explains the prominent use of assimilation during diagnosis and treatment stages for participants in this study.

#### Subtheme 2c: Finding purpose in impairment

All participants were able to find purpose in their impairment, which enabled an acceptance of and adaptation to their impairment. This process was facilitated by coping strategies such as accepting the uncontrollable and positive reframing. Participants spoke of their impairment largely as a side note, or an afterthought, to their main story. Participants who were cancer-free framed their impairment as a reasonable and tolerable consequence of surviving cancer. Numerous participants framed the treatment as having “done its job”. They expressed gratitude at having survived cancer, with the impairment standing as an ongoing reminder or symbol of their survival and the efficacy of treatment. In this way, participants appeared to attribute purpose to their impairment, and whilst the impairment was not necessarily embraced or desired, it was accepted, tolerated, and carried embedded meaning. Finding this purpose made the ongoing impairment more tolerable and brought a sense of coherence and resolution to their experience, which minimised incongruence and distress. This experience is consistent with meaning-focused coping, which involves drawing on beliefs, values, and existential goals to motivate and sustain coping. Positive reappraisal is a key aspect of meaning-focused coping and involves a process of re-evaluation and reinterpretation of the stressful experience to derive new meaning and sustain wellbeing. Meaning-focused coping tends to be drawn upon in instances where there are limited possibilities for major changes to the objective features of the situation [34], such as coping with ongoing impairment.

Although increased distress has been associated with the experience of treatment-related impairment in other studies [11, 35], there was not notable distress surrounding impairment for participants in this study who had experienced PTG. Given that participants faced the task of coping with ongoing impairment later in their cancer trajectory, this suggests that for most, the process of coping with diagnosis and treatment was established, so their experience of impairment was more readily assimilated.

Participants reflect upon the meaning they attributed to impairment with acceptance and restored congruence (Participants 1 and 5, Table 6), with the impairment itself sometimes symbolic of active treatment and recovery (Participant 2, Table 5). For one participant, the physical changes occurred early in her cancer experience, and she could readily attribute adequate and positive meaning to her impairment due to the resolution of symptoms. The “invisible” nature of changes also aided in her positively interpreting them, with the hysterectomy eliminating risk and uncertainty, providing a sense of stability, empowerment, and hope as she faced further treatment (Participant 6, Table 6).

**Table 6:** Substantiating quotes relating to finding purpose in impairment

Participant	Aspect of incongruence/subtheme	Substantiating quotes
1	Acceptance and restored congruence facilitated attribution of meaning to impairment	*They told me, “it could last a week, a month, six months or 12 months, or it could be with you forever”. The way I look at it, it’s going to be forever now…You just deal with it, that’s all…. I haven’t worried about it. You don’t like it. The way I was looking at it was, the treatment was doing its job and so far, the results are right*
5	Acceptance of impairment, weighed up with remission	*I’d have the radiation morning and night, and it did its job. I haven’t had the cancer back since, but there are a lot of side effects due to the double doses….., I can live with what, I can accept what I’ve got and just get on with life and no good stressing out and saying “if things had been different”, you’ve just got to accept it*
2	Reciprocal and symbolic nature of attributed meaning	*If I’ve got neuropathy for the rest of my life that’s fine as long as the chemo has done its job, and I can deal with it. I’ll work around that. I’ll do what I can do to help it*
6	Positive concept of impairment from symptom resolution	*Because I’ve had a hysterectomy, I don’t have any hormonal issues, I don’t have any bleeding. I don’t worry about any of that stuff now. So in a sense, you know, in one sense I actually feel better than what I did before*
6	‘Invisible’ nature of physical changes supported positive interpretation of impairment	*I didn’t feel any different because for me the, you know, the uterus and stuff was like inside and so outwardly nothing had changed. If I would’ve lost a leg or an arm I would’ve felt much more, you know, like I’d been through something or, and something had seriously changed, or somehow I was different*
3	Meaning making involved redefining life purpose and goals	*Well, just by putting my thoughts and my mind into doing everyday normal things, like getting out of bed and getting dressed. I’m just grateful that I can still so all of that. Yeah, I don’t know which is the best way to explain that, but anyhow, it doesn’t make me anxious now, because I’ve learned to cope with that*
4	Daily process of adaptation and redefining goals	*I’m used to getting up in the morning and saying “I’ve got six things to do, I’ll do them”. I had to revisit that and say,“well listen, I’ve got six things to do but I’ll probably only do one because of that fatigue level”. That was hard to adjust to*

For participants living with incurable cancer, making meaning of their impairment involved redefining their purpose and goals in life which brought a sense of resolution and gratitude for their enduring capacities and functioning (Participant 3, Table 6). Some came to embrace their “new” self, making clear links between vulnerability, adaptation, and PTG. Whilst he experienced fundamental and far-reaching PTG outcomes, one participant reported the daily process of adapting and redefining goals, as he attempted to process and establish new expectations of himself (Participant 4, Table 6).

This study demonstrated the distinction between the meaning making process and the outcomes of that process. In their study on meaning making and adjustment following cancer, Park et al. make the same distinction, framing PTG outcomes as “meanings made” [36]. The process of meaning reconstruction is thought to result in effective adaptation if sufficient meaning is found. However, if survivors are not able to assimilate their cancer experience into existing belief systems or accommodate their beliefs in consideration of their cancer experience, this process can take the form of unhelpful rumination and lack of resolution. As reflected in the current study, growth occurs when individuals can constructively resolve the search for meaning [37, 38] explored post-treatment challenges impacting upon the quality of life of rural cancer survivors and suggest that quality of life is often not restored, and negative impacts persist beyond stages of treatment, including ongoing physical and psychological effects. Although finding adequate meaning in their experience of impairment was attained by participants in the current study, this is by no means an expected outcome for cancer survivors. For example, van der Spek et al. (2013) explored meaning making in cancer survivors and found that cancer survivors generally experience increased meaning after cancer in at least one aspect [29]. However, in instances where loss of meaning was also experienced, this was often in relation to physical impairments with participants experiencing difficulty reconciling their changed self and physical limitations post-diagnosis.

### Theme 3: The legacy of coping with cancer

The influential factor that facilitated participants to move beyond equilibrium and achieve PTG was their ability to utilise *accommodation* to achieve congruence, by altering pre-existing beliefs and assumptions to fit in with their new experiences. Participants integrated their impairment into their *post-cancer identity* and achieved a sense of resolution and congruence associated with their *ongoing impairment*. This may be attributable to the fact that, for the majority, recognition that impairment is likely to be ongoing emerged in the latter stages of their cancer trajectory. It is theorised that at this point, their process of meaning making was well established, and therefore the *legacy of coping* with cancer was more readily assimilated.

#### Subtheme 3a: Multi-dimensional growth: accommodating new meaning

The capacity of participants to accommodate new meaning was a key factor in promoting PTG in this study. Although it was also central to the meaning making process, accommodation was most evident in participant reflections on PTG outcomes. Participant reflections on growth demonstrated how they adjusted their worldview and self-concept to incorporate new meanings from their experience of cancer. While distress can be eased through assimilation or accommodation of new cancer-related information, to progress beyond the pre-cancer baseline demands accommodation, given that growth is fundamentally about the development of new worldviews [31]. The domains of PTG experienced by participants included new possibilities, relating to others, personal strength, and appreciation of life, with spiritual change not a feature in this cohort [5].

Growth in the domain of new possibilities encompassed changed priorities, new perspectives on challenges, and a desire and intention to maintain growth outcomes into the future. These aspects of growth were experienced as prioritising one’s own health and wellbeing, prioritising family time and leisure over business commitments and financial gain, reduced concern over relatively minor problems, embracing life’s everyday stressors, and purposefully applying their learnings and growth to guide decision-making. Insights gained from experiencing cancer changed priorities, creating space for opportunities and openness to new experiences (Participant 2, Table 7). This new perspective was characterised by deepened inner strength and wisdom, which brought renewed appreciation for normality and a sense of comfort in being able to focus on the routine challenges of life (Participant 6, Table 7).

**Table 7:** Substantiating quotes relating to subtheme of multi-dimensional growth through accommodating new meaning

Participant	Aspect of incongruence/subtheme	Substantiating quotes
2	New possibilities	*My husband got offered a job which was going to be more hours, more weekends…., and previously we probably would be like, “yeah, do it, that’s great”. But then that means that things we’ve taken out of this go behind us. So I really just think it’s just about making time for what you love and who you love and not …. worrying so much about the little things*
6	Inner strength, wisdom and everyday life appreciation	*Then it just puts other things into perspective too,…..I would love nothing more than to be back at work and….stressed out with dumb work stuff because by comparison that just felt so….trivial compared to the cancer journey that I went on*
8	Increased empathy, vulnerability and personal growth in relating to others	*More compassionate. More understanding. Not being so distant with people*
4	Recognising vulnerability in self and others resulted in finding mutual benefit in giving and accepting support	*Probably since I’ve had cancer I haven’t been so remote…if I hadn’t got cancer I don’t know what would have happened between us. We probably would have stayed married….* *I mean, now I’m prepared to listen to other people. I was going to go down and….offer to help say, Salvation Army or those places to help people who are in financial problems…But I wouldn’t have even thought about that a few years ago. I would have thought “that’s their problem, let them deal with it” whereas, yeah, that’s how I changed*
7	Willingness to hand over some responsibility	*Probably as a mother, as a teacher, as whatever, you are in control of a lot of things and I’ve just had to say, well, I’ll have to let others be in control. Sometimes, it niggles, but mostly, I’ve accepted it now and it is an acceptance*
6	Overcoming challenges instilled awareness of own inner strength and resilience	*We can’t avoid the trauma and be resilient, I think we need to go through the trauma to then realise these are the things that we do to get through it and now…. I recognise my resilience and I really feel that that’s what happened*
7	Self-belief enabled ownership and self-belief	*I’ve had to do this all alone virtually and that’s something that I’ve proved to myself that I could do, whereas previously with all the other things, (my late husband’s) always been there and the kids have been there*
3	Adaptability in response to unprecedented adversity	*Well, everything’s changed, because I was a full-on person doing everything, going to a person that had to be grounded… I just learned to cope with what my boundaries were…* *I’ve always been inspired by life, and I’ve always tried to do what he challenges are, but then when I found out that I couldn’t do many of the harder things, I can do the easy things*
4	Physical and psychological adaptation	*You can’t be the person you used to be. As I said, I was a person where everything I did I did really well and now everything I do, I do pretty badly, except photography!*
5	Patients derived from future uncertainty and appreciation of present	*Yeah, because before this I just took life too cheap and what was I saying, I used to say “I work to live, not live to work”. So, in other words, fight life, get out and enjoy life all the time, but now, I’m very careful……I’d always go over the limit, but now I think, “what’s the use?”. I try to do the right thing behind the wheel. Try and do everything, except me few beers of course…, instead of going like a bull at a gate*
8	Cancer demanded capacity for patience	*I’m probably more accepting of things than what I used to be before, more patient I guess, more patient, you’ve got to be patient when you’ve got this blessed thing. You’ve got to be patient. Nothing’s rushed, you can’t rush things*

Growth in the domain of relating to others incorporated increased empathy, changes to relationships, assertiveness, and help-seeking capability. Increased empathy was experienced by many participants and emerged from exposure to circumstances beyond one’s direct control. Participants described how this growth involved acknowledgement of prior naivety and good fortune, recognition and acceptance of vulnerability in self and others, and for participant 8, his cancer-related distress brought deeper compassion for others (Participants 4 and 8, Table 7).

Relationship changes included deepening connections, increased openness to relationships and connection with others, greater understanding of one’s own relationship needs and priorities, and valuing reciprocal relationships. Participant 4 experienced multi-faceted growth in the domain of relating to others, including increased openness, a newfound willingness and ability to connect with others, empathy, altruism, and help-seeking capacities. He described himself as previously self-focused, detached, and hardened by life’s adversity, and his “new” self as “softened”. Recognising his own vulnerability and vulnerabilities in others led him to find mutual benefit in both supporting others and accepting support. This growth resulted in deeper connections with others, including a transformative influence on his relationship with his wife (Participant 4, Table 7).

Participant 7 identified increased help-seeking capacities, or “*the willingness to let others take control*” as her most profound aspect of growth. Her previous life roles involved a sense of control, influence, and responsibility, and she recognised that handing over responsibility to others was a necessary adjustment throughout her cancer experience (Table 7).

Growth in the domain of personal strength was experienced as greater adaptability, enhanced self-belief, increased patience, and acceptance. Participants also experienced enhanced appreciation for their coping capabilities, as cancer brought unforeseen challenges. The confrontation and overcoming of these challenges brought increased awareness to their own inner strength with participants describing enhanced resilience and self-efficacy. Participants also described a trust and confidence in their ability to apply this strength to future challenges (Participants 6 and 7, Table 7).

Adaptability was evident as a PTG outcome as well as being an important aspect of the meaning making process. As a result of coping with their prognosis and impairment, participants with incurable cancer demonstrated increased adaptability in response to unprecedented adversity. This adaptability extended beyond adjusting to their physical limitations to a shift in mindset, their approach to daily life, and how they viewed themselves. They were able to frame adaptations as meaningful and embrace their current selves and circumstances with acceptance and positivity, rather than simply tolerating these changes (Participant 3, Table 7), adapting physically and psychologically to changes necessitated by their cancer experience (Participant 4, Table 7).

Patience was derived from acknowledgement of the fragility or uncertainty of the future and increased appreciation for the present moment. This allowed participants to be more mindful and self-compassionate, to reflect philosophically about their behaviours and priorities. Participants also became more aware of the value of patience as a virtue, expressing a desire to embody patience in their daily lives. For participant 5, newfound patience allowed him to balance passion and responsibility, with greater consideration of health and safety consequences (Table 7). Participant 8 reflected on the way cancer demanded of him both patience and acceptance, a capacity which had been translated into daily life post-cancer (Table 7).

Greater appreciation of life was embedded within participants’ experiences and described in both explicit and implicit ways. Participants described the insights they had gained through coping with their cancer experience, which brought diverse and unprecedented challenges. Through the process of PTG, participants came to experience life and view themselves in new ways. Greater appreciation for life was embodied in participant reflections on new possibilities for the future, changed priorities, increased self-awareness and self-efficacy, greater sense of purpose, and connection with others. Participants also explicitly detailed a greater appreciation of life, expressing deeper gratitude and wisdom, recognition of life’s fragility, and the indiscriminate nature of cancer. Participants spoke of relishing a return to normality, finding comfort in previously mundane routines following the unpredictability and chaos of cancer (Participant 5, Table 7).

Growth was most common in the domains of “personal strength” and “relating to others”, with “spiritual change” not described by participants. These findings are consistent with other Australian studies, which report that the domains of PTG most reported within Australian populations are “relating to others”, “appreciation of life”, and “personal strength”. Spiritual change has not been prominent in Australian’s PTG experiences [16]. Many participants experienced growth across multiple domains, highlighting the multi-dimensional nature of PTG [14].

#### Subtheme 3b: Post-cancer identity

Two participants reported significant growth to the extent that these positive changes constituted the development of distinct post-cancer identities. They both experienced breadth and depth of growth, occurring across four domains, and provided insightful, specific descriptions of their growth. Both had utilised a wide range of coping strategies to cope with their cancer experience. However, interestingly, these two participants had distinct demographic and illness characteristics, which prompted the analysis and reflection from which the eventual theoretical model emerged. Participant 2 was a younger female, who had been diagnosed with early-stage cancer and experienced peripheral neuropathy. She was cancer and treatment free at the time of interview. She had experienced high distress associated with her cancer diagnosis and reported ongoing fear of recurrence. In contrast, participant 4 was an older male, who lives with incurable cancer and ongoing treatment. Despite enduring significant cancer- and treatment-related impairment, his distress was less prominent. Consistent with the overall study findings, the common factor between the two participants appears to be the experience of high incongruence which stimulated a high level of active rumination and critical reflection for these participants. This meaning making process culminated in the development and embracing of a new worldview and self-concept. The meaning making process of participant 2 was indicative of self-discovery. Her reflections demonstrate the development of a broader and more critical view of self and the world in response to unexpected and unparalleled adversity. This process involved re-evaluating how she viewed the world, herself, and her place in it, leading to growth outcomes including increased patience, assertiveness, empathy, and changed priorities (Participant 2, Table 8).

**Table 8:** Substantiating quotes relating to post-cancer identity

Participant	Aspect of incongruence/subtheme	Substantiating quotes
2	Self-discovery	*Well I feel like I was probably a bit sheltered prior. Just you know, I lived my life, and we have a good life. I just kind of was like, “that’s really horrible but that’s never going to happen to us. That’s not going to happen to me”*
4	Redefined self-identity	*That helped a lot to try and say to myself “this is the old (me), this is the new one, and the new one is a very different person that the old one who would get up there….”*
	Redefined longstanding worldview	*I always thought that nothing could touch me…..But now it’s a different thing, it’s going to be a slow death basically. I think I start to think about that, and I think about all the other people that are going through the same thing and ….., seeing those young kids that have got cancer, that sort of changed me. I started to see people in a different light*

The meaning making process of participant 4 involved redefining his long-established world view and self-identity, prompted by his cancer experience leading to recognition of vulnerability. Growth outcomes included changed priorities, increased openness, connection and empathy, increased help-seeking, and adaptability (Table 8).

The notion of post-cancer identity has been alluded to previously. Mazariego et al. found the participants’ post-cancer identity included a renewed role in and commitment to personal relationships [3]. Newfound empathy and altruism for others with cancer and a new sense of identity were reported by Threader and McCormack [13].

#### Subtheme 3c: Ongoing impacts: “The burden you carry with you”

Experiencing PTG did not preclude participants from experiencing ongoing adverse effects, including the physical impacts central to this study. Participants acknowledged the simultaneous burden of impairment and being able to find purpose in their experience (Participant 7, Table 9) or to find acceptance and recognise their progress (Participant 2, Table 9). The experiences of participants also reflected the notion that the psychological impacts of cancer endure beyond the treatment stage, where being free from cancer by medical definition does not necessarily equate to being free from the impact of cancer. The psychological impact can be ongoing, on top of the ongoing physical impairments experienced by participants. Several participants spoke of a sense of wanting to contain their cancer experience to the past, while acknowledging its ongoing presence, as they described how cancer intrudes into both their present and future. For some, this fear or acknowledgement was a relatively passive presence (Participant 7, Table 8). For others, the fear was a key factor in their experience of PTG which drove their desire to sustain the personal changes and psychological growth gained through coping with cancer (Participant 2, Table 9).

**Table 9:** Substantiating quotes relating to ongoing impacts of cancer

Participant	Aspect/subtheme	Substantiating quotes
7	Ongoing adverse effects, including physical impacts	*I think it’s (the impairment) a bit more of a burden, but it has taught me to just bide my time and do more research*
2	Burden balanced with acceptance	*Doing a bra up is still a bit tricky but I’m definitely much better. But it’s [peripheral neuropathy] a constant, it’s not a thing that comes and goes. It’s in my hands and feet and that’s just what it is*
7	Ongoing psychological impacts	*…..but the cancer is always sitting in the back of my mind*
2	Fear can drive desire to sustain growth outcomes	*The biggest thing I feel like is, one of the things I keep saying. If it comes back, which touch wood it never does, I want to know that I’ve done everything in my power to make sure that it doesn’t*
2	Fear balanced by understanding	*I was scared. And I’m still scared but I have more of an understanding*
6	Surveillance reignites uncertainty and distress	*It’s like a burden that you carry with you, and I just …think that’s just the reality of it. I don’t see that, you know, there’s no way you can avoid it, you have to go through that and work through it however you do*
6	Incongruence of feeling well and fear of recurrence	*oh, it’s pretty horrid. It’s every three months when those tests come up …….because I feel perfectly healthy in between the three months I almost forget, you know, just I live normally, I’m not sick, I’m not having to come to hospitals…Then the time comes and you see it in your calendar and you do feel that sort of nervousness….just that worry, you’re like, no, no, no, just calm down, and very much that trying to keep calm and do that mindfulness stuff and…just don’t let your brain go down that rabbit hole, and just stay sort of positive and stay focused, so far so good, you know… I guess some months might be harder than others if you’ve got other stuff going on, or, you know, you feel more vulnerable*
8	Avoidance to cope with ongoing distress	*I don’t look back. I don’t want to go back to where I was. I know I said the other day, “don’t talk about it”. Because if you talk about it, it only brings back bad memories, and it does……I just want to move forward and forget about things, you know? It’s been a tough time. Yeah. Just wash it out of your soul a little if you can*

For participants who were now living cancer-free, surveillance was still accompanied by some distress, largely stemming from feelings of uncertainty. Ongoing distress and unease were framed as an inevitable and accepted consequence of surviving cancer (Participant 6, Table 9). This participant also described a sense of incongruence about feeling well yet having the fear of recurrence looming over her, which necessitated the ongoing use of coping strategies to manage. Participant 8 described the ongoing use of avoidance to cope with cancer-related psychological distress (Table 9).

This theme is reflected in Tedeschi and Calhoun’s functional-descriptive model which acknowledges that some distress may persist beyond the process of meaning making, albeit at a lower level than experienced in the initial stages following trauma [31]. An influential theory of growth following trauma, the organismic valuing theory by Joseph and Linley [31], acknowledges that motivation towards growth following trauma is inherent given an enabling social environment, and occurs via accommodation. However, accommodation does not necessarily result in positive or adaptive changes in worldviews. It is dependent upon the meaning ascribed to the trauma, which is influenced by several personality, psychological, social, demographic, and situational variables.

### Strengths and limitations

The qualitative nature of this study captures the depth and subjectivity of the lived experiences of cancer survivors, and the interpretive approach to understanding these experiences is acknowledged and explained. Although the study does not aim for or claim objectivity or generalisability, it cannot draw conclusions about causal relationships between treatment-related impairment and PTG. Despite this, the diversity of the sample in terms of illness and demographic characteristics was a strength of this study, including variety in age, gender, and tumour stream. The inclusion of participants with metastatic/incurable cancer was valuable, as this cohort of participants is commonly excluded from other studies. The criteria of having experienced “self-perceived PTG” through coping with cancer remain valid and consistent with the research methodology and aims.

## Conclusion

People with ongoing cancer treatment–related impairment can experience PTG as a coping process and as an outcome of dealing with adversity. The extent of PTG in cancer survivors may depend on the degree of incongruence they experience and their ability to accommodate these contradictions to develop new meaning. This research highlights the distinction between coping with cancer treatment and the concept of PTG, while also acknowledging that PTG does not preclude survivors from experiencing ongoing distress, although possibly at reduced levels. The implications from this study include the need for awareness and dialogue about the potential for positive psychological outcomes following trauma and adversity, that finding adequate meaning in treatment-related impairment can enable acceptance and adaptation, and that previous life experiences of adversity are influential in the experience of PTG. Further research is needed to understand how early or visible treatment-related impairment influences cancer survivors’ PTG.

## Data Availability

Participants did not consent for secondary use of data, which is therefore not available for circulation.
